# Detection of anti-*Leishmania* spp. antibodies in poultry from central region of Rio Grande do Sul, Brazil

**DOI:** 10.1590/S1984-29612023077

**Published:** 2023-12-08

**Authors:** Maurício Tatto, Fagner D’ambroso Fernandes, Eliesse Pereira Costa, Fabio Yuji Shibuya, Luiza Isaia de Freitas, Vanessa Osmari, Isac Junior Roman, Patrícia Bräunig, Fernanda Silveira Flores Vogel, Sônia de Avila Botton, Luis Antônio Sangioni

**Affiliations:** 1 Laboratório de Doenças Parasitárias, Departamento de Medicina Veterinária Preventiva, Universidade Federal de Santa Maria – UFSM, Santa Maria, RS, Brasil; 2 Centro Universitário Ritter dos Reis – UniRitter, Porto Alegre, RS, Brasil; 3 Laboratório Central de Diagnóstico em Patologias Aviárias, Departamento de Medicina Veterinária Preventiva, Universidade Federal de Santa Maria – UFSM, Santa Maria, RS, Brasil; 4 Laboratório de Saúde Única, Departamento de Medicina Veterinária Preventiva, Universidade Federal de Santa Maria – UFSM, Santa Maria, RS, Brasil

**Keywords:** Domestic birds, antibodies, indirect immunofluorescence reaction, leishmaniasis, Aves domésticas, anticorpos, reação de imunofluorescência indireta, leishmaniose

## Abstract

Domestic birds such as *Gallus gallus*, *Meleagris gallopavo*, *Anser anser* and *Numida meleagris* are widely distributed throughout the world and maintain contact with humans and other animal species considered reservoirs of both Visceral Leishmaniasis (VL) and American Tegumentary Leishmaniasis (ATL), including dogs and cats; wild canids, marsupials; and synanthropic animals such as rodents and chiroptera. Therefore, this study aimed to detect the presence of anti-*Leishmania* spp. antibodies in birds from a rural area of the municipality of Santa Maria, southern Brazil. From May to December 2022, 262 blood samples were collected from 244 chickens, 8 turkeys, 7 guinea fowl and 3 geese, distributed in 27 rural properties in 6 districts. All the sites visited presented positive birds for the presence of *Leishmania* spp. Thus, it is inferred that, contact with this protozoan can induce the production of antibodies, suggesting that these animals can be used as sentinels for the circulation of this agent. In addition, the blood of these animals is a preferred food source for insects of the subfamily Phlebotominae, which can be used them as bioindicators of the presence of these phlebotomes.

## Resumo

Aves domésticas, como *Gallus gallus*, *Meleagris gallopavo*, *Anser anser* e *Numida meleagris,* são amplamente distribuídas pelo mundo e mantêm contato com humanos e outras espécies animais consideradas reservatórios tanto da Leishmaniose Visceral (LV) quanto da Leishmaniose Tegumentar Americana (LTA), incluindo-se cães e gatos, canídeos silvestres, marsupiais; e animais sinantrópicos, como roedores e quirópteros. Portanto, este estudo teve como objetivo detectar a presença de anticorpos anti-*Leishmania* spp. em aves de uma área rural do município de Santa Maria, sul do Brasil. De maio a dezembro de 2022, foram coletadas 262 amostras de sangue de 244 galinhas, 8 perus, 7 pintadas e 3 gansos, distribuídas em 27 propriedades rurais de 6 distritos. Todos os locais visitados apresentaram aves positivas para a presença de *Leishmania* spp. Assim, infere-se que o contato com esse protozoário pode induzir a produção de anticorpos, sugerindo que esses animais possam ser utilizados como sentinelas para a circulação desse agente. Além disso, o sangue desses animais é uma fonte de alimento preferencial para insetos da subfamília Phlebotominae, que podem ser utilizados como bioindicadores da presença desses flebotomíneos.

## Introduction

Domestic birds, such as chickens (*Gallus gallus domesticus*), turkeys (*Meleagris gallopavo*), geese (*Anser anser*) and guinea fowl (*Numida meleagris)* are globally distributed animals that are raised and kept in peridomestic spaces, such as backyards, both in urban and rural areas. These birds keep close contact with humans and various domestic animals, such as dogs (*Canis familiaris*) and cats (*Felis catus*), wild predators like foxes (*Dusicyon vetulus* and *Cerdocyon thous*) and marsupials (*Didelphis albiventris* and *Didelphis marsupialis),* and synanthropic animals such as murines (*Rattus rattus)* (Sherlock, 1996; Alexander et al., 2002; Ratzlaff et al., 2023) and chiropterans, which some studies found infected by some *Leishmania* species, indicating that bats may participate in the biological cycle of *Leishmania* spp. and act as a host, reservoir, and/or source of infection (Savani et al., 2010; Shapiro et al., 2013; Ratzlaff et al., 2022).

These birds are in contact with several blood-sucking insects that are vectors of these protozoa, such as Phlebotominae (Diptera: Psychodidae: Phlebotominae), and transmitters of leishmaniasis in both humans and animals. According to the literature review conducted by Sousa et al. (2021), chickens were among the seven animals that most participated in the feeding pattern of sandflies in Brazil.

Consequently, chickens may favor a link between wild and domestic disease transmission cycles (Dantas-Torres & Brandão-Filho, 2006).

However, in addition to sandflies, there are records of other ornithophilic hematophagous dipterans naturally infected by protozoa of the family Trypanosomatidae, mainly avian trypanosomes (Svobodová et al., 2015). Srisuton et al. (2019) found that sandflies may be potential vectors of *Leishmania* spp. and *Trypanosoma* spp. in southern Thailand, including concomitantly. In Brazil, infections by protozoa of the genus *Trypanosoma* have been recorded in several species of sandflies, but few parasites have been isolated and characterized (Shaw et al., 2003). In addition, there are several reports of birds infected with trypanosomatids of the genera *Herpetomonas*, *Crithidia* and *Leptomonas* (Lukeš et al., 2014).

Chickens have physiological characteristics, such as high body temperature (41 ºC), which prevents the multiplication of protozoa in these hosts, protecting them from disease. However, their shelter is considered a place of rest and reproduction for Phlebotominae, which is relevant for maintaining and multiplying vector populations (Nieves et al., 2011). In addition, poultry farms can increase the risk of contracting leishmaniasis by up to four times compared to households without chicken coops (Rodrigues et al., 1999).

In this sense, Borges et al. (2009) highlight the epidemiological importance of domestic chickens in the leishmaniasis transmission cycle, owing to their potential to generate a favorable environment for the development of Phlebotominae.

It should be noted that Visceral Leishmaniasis (VL) and American Tegumentary Leishmaniasis (ATL) affect the most vulnerable human populations with restricted access to health services. In Brazil, VL is caused by protozoa of *Leishmania* (*Leishmania*) *infantum* species and is transmitted by Phlebotominae of the species *Lutzomyia longipalpis* and *Lutzomyia cruzi* (Queiroz et al., 2012; Brasil, 2014). Other species, such as *Nyssomyia neivai*, *Pintomyia fischeri,* and *Migonemyia migonei*, are also implicated in the infection (Salomón et al., 2010; Carvalho et al., 2010; Dias et al., 2013; Guimarães et al., 2016).

The first autochthonous cases of the disease in Rio Grande do Sul occurred in dogs in 2008 and humans in 2009, in the municipality of São Borja, on the western border of the state (Souza et al., 2009; CEVS, 2011).

In Santa Maria, suspected cases disease has been reported in dogs since 1985 (Pocai et al., 1998). However, from 2017 onwards, there was an increase in the cases of canine visceral leishmaniasis (116 cases), and in 2021, two autochthonous human cases were recorded, one of which contributed to the patient's death (Diário de Santa Maria, 2021a, b).

In a study performed by Osmari (2023), the author confirmed the presence of three species of vectors of importance for public and animal health, being the first report of the presence of *L. longipalpis* (Osmari et al., 2022), which is considered the primary vector of VL in Brazil by the Ministry of Health. In addition, *P. fischeri* and *M. migonei* are also implicated as transmitters of the visceral and mucocutaneous form of the disease (Rêgo et al., 2020).

Considering the epidemiological importance of leishmaniasis and the scarcity of research on *Leishmania* spp. infection in birds, this study aimed to detect the presence of anti-*Leishmania* spp. antibodies in poultry in a rural area of the municipality of Santa Maria, southern Brazil.

## Materials and Methods

**Figure 1:**
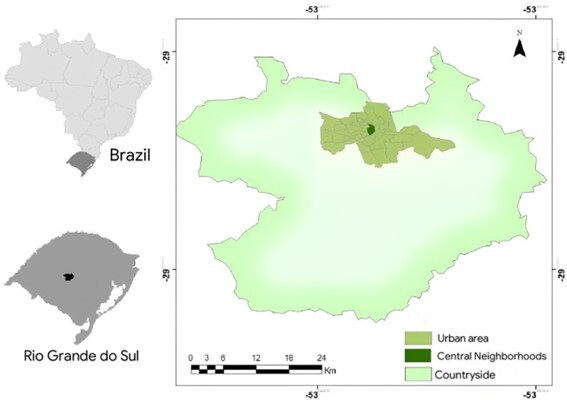
Study site map [adapted from Schiavo et al. (2016)].

The municipality of Santa Maria is in the central region of the state of Rio Grande do Sul (RS) in southern Brazil, where the Atlantic Forest and Pampa biomes predominate. The city has an area of 1,780.194 km^2^, an estimated population of 296,081 inhabitants, a humid subtropical climate, and an average annual temperature of 19.4 °C (IBGE, 2021) (Figure 1).

According to data provided by the Epidemiology and Statistics Section of the Department of Agriculture, Livestock, and Rural Development of Rio Grande do Sul, Santa Maria has 5021 chickens, 42 guinea fowl, 27 geese, and nine turkeys. Rural producers in the region provided this information during the 2021 annual herd declaration.

In this study, samples were collected according to the availability of those responsible for the animals. Between May and December 2022, 262 blood samples were collected from 244 chickens, eight turkeys, seven guinea fowl, and three geese distributed across 27 rural properties in six districts of the municipality of Santa Maria, Rio Grande do Sul, Brazil.

Blood was collected from the birds by restraining, cleaning the skin, using hydrophilic cotton and alcohol, followed by puncture of the medial ulnar vein or through the most viable vessel during handling, using sterile 3 mL syringes with disposable hypodermic needles of 26 G (0.45 x 13 mm), with a maximum volume of 2.5 mL collected per animal, proportional to live weight. The blood was stored in collection tubes without anticoagulant at a temperature between 3º and 8 ºC, stored in isothermal boxes, and sent to the Laboratório de Doenças Parasitárias the Universidade Federal de Santa Maria (LADOPAR-UFSM). Subsequently, the samples of blood were centrifuged at 1600 x g for 10 min to obtain the serum. The serum samples stored at -20 ºC until the Indirect Immunofluorescence Reaction (IFA) was performed.

For the detection of anti-*Leishmania* spp. antibodies, IFA was performed using 12-well slides sensitized with *L. infantum* promastigotes, which were used as antigens to detect immunoglobulin Y (IgY). As a secondary antibody, IgY (Affinity Purified Antibody Fluorescein. Sigma, St Louis, MO, USA) was used as an anti-chicken antibody conjugated with fluorescein, at dilution 1:100, according to the technique described by Otranto et al. (2010). Positive and negative samples were obtained from the serum bank of LADOPAR-UFSM and used as controls. All positive serum samples in the 1:30 dilution was considered reactive according to the same author, and were subsequently diluted to determine the maximum antibody titer. After conducting the tests, the results were tabulated on a spreadsheet, and descriptive analyses were performed using Microsoft Excel.

To minimize the influence of cross-reactivity between trypanosomatids, IFA was performed according to Camargo & Rebonato (1969), for detection of anti-*Trypanosoma* spp. antibodies in those reagent samples for *Leishmania* sp., using IgY (Affinity Purified Antibody Fluorescein. Sigma, St Louis, MO, USA) as a secondary antibody at dilution 1:100.). Positive and negative samples were obtained from the serum bank of LADOPAR-UFSM and used as controls. All positive serum samples in the 1:30 dilution was considered reactive according to the same author, and were subsequently diluted to determine the maximum antibody titer.

## Results

All locations visited had reactive birds with IgY anti-*Leishmania* spp. antibody titers. Of the 244 blood samples collected from the chickens, 181 (74.2%) were reactive presenting titles ranging from 30 to 960 (Table 1). On the other hand, no reactive sample for *Leishmania* spp. was positive for *Trypanosoma* spp.

**Table 1:** Frequency of anti-*Leishmania* spp. antibodies detected through the indirect immunofluorescence reaction in chickens reared extensively in six rural districts of Santa Maria/RS, from May to December 2022.

**Location (number of properties)**	**Antibody Titles**						**Reagent samples/Total (%)**
	**30**	**60**	**120**	**240**	**480**	**960**	
Palma (2)	3	4	3	4	0	0	14/22 (63.6%)
Arroio Grande (5)	7	4	9	11	0	0	31/43 (72.1%)
Arroio do Só (5)	6	3	14	15	2	1	41/50 (82%)
Painss (4)	5	7	11	7	0	0	30/38 (79%)
Passo do Verde (6)	3	7	12	4	0	3	29/44 (66%)
Santa Flora (5)	11	7	9	8	1	0	36/47 (76.6%)
Total (27)	35	32	58	49	3	4	181/ 244 (74.2%)

Among the locations where the study was performed, the district of Arroio do Só had the highest number of reactive samples, although the results remained proportional to the number of samples collected in each location.

Three Geese and six turkeys showed reactive tests for 30 and 60 antibody titers, respectively; three turkey samples were still reactive for 120 anti-*Leishmania* spp. antibody titers. Both species were bred in the Palma District (Table 2).

**Table 2:** Frequency of anti-*Leishmania* spp. antibodies detected by indirect immunofluorescence reaction in turkeys and geese reared extensively in six rural districts of Santa Maria/RS, from May to December 2022.

**Animal species (number of animals collected)**	**Antibody Titles**		**Location**
	**60**	**120**	
Turkeys *(Meleagris gallopavo)* (8)	3	3	Palma
Geese *(Anser anser)* (3)	2	1	Passo do Verde

## Discussion

Several studies have attempted to elucidate the role of domestic birds in leishmaniasis infection. These animals are raised worldwide as protein sources, either for the production of meat or eggs, mainly on rural properties, and are raised by the most vulnerable populations with restricted access to health services.

In Brazil, Alexander et al. (2002) summarized that the role of domestic birds in the epidemiology of VL involves a balance between zooprophylaxis, maintenance of Phlebotominae populations, and attraction of predators that are reservoirs and hosts of *L. infantum*. However, until now, *Leishmania* sp. infection has not developed in birds (Dantas-Torres & Brandão-Filho, 2006).

This occurs owing to factors that have been suggested by Alexander et al. (2002), such as a body temperature higher than that of mammals (41 and 37 ºC) and nucleated erythrocytes that stimulate DNAase activity, which can be fatal for *Leishmania* spp. in the gut of sandflies, among others.

The IFA technique is based on the use of the intact parasite as an antigen and is very useful in epidemiological studies, as its sensitivity varies from 83 to 100% and its specificity is approximately 80% for serum samples (Bresciani et al., 2008). This test has been used to demonstrate contact between host and parasite with very high sensitivity (Desquesnes et al., 2022).

However, the overlapping of areas where closely related etiological groups are present, such as parasite species belonging to the Trypanosomatidae family, is a limiting aspect of the interpretation of serological data due to possible cross-serological reactions caused by phylogenetic proximity, which can give false results. positive (Caballero et al., 2007).

The family Trypanosomatidae (phylum Protozoa, class Kinetoplastida) comprises: 14 monoxenic genera that infect insects (e.g. *Leptomonas* spp., *Herpetomonas* spp.) and five dixenic genera, with invertebrates as vectors (genus that infects Phytomonas plants), and another four that infect animals and humans (Desquesnes et al., 2022). Although most representatives of this family are not pathogenic for their hosts, some are important etiological agents of human and domestic animal diseases, such as African and American trypanosomiasis (Chagas disease) and leishmaniasis (Simpson et al., 2006).

Otranto et al. (2010) tested 121 serum samples from poultry, including 34 domestic geese seven wild ducks (*Cairina moschata*), five pheasants (*Phasianus colchicus*), two guinea fowls and 73 chickens, for the presence of anti-*L. infantum* antibodies by indirect immunofluorescence testing. At the time, all 73 serum samples from chickens were negative in IFA, while three serum samples of *A. anser* and one *P. colchicus* were positive with titers ranging from 30 to 60.

In our study, it was also possible to detect anti-*Leishmania* sp. antibodies in the three geese, and 6 turkeys, in addition to 181 chickens, totaling 190 (74.5%) reactive animals, with titers ranging from 30 to 960. On the other hand, none of these samples obtained the same result when we aimed to detect anti-*Trypanosoma* sp. antibodies, which minimized the limitation imposed by the possible cross-reaction between the genus *Leishmania* and *Trypanosoma*.

These results indicate a high rate of anti- *Leishmania* spp. antibodies in these animals. Therefore, this study can be characterized as the first report of *Leishmania* spp. antibodies in chickens, turkeys and geese raised extensively in the central region of RS and additional more specific diagnostic tests such as polymerase chain reaction (PCR) are necessary to confirm the species to which these animals are exposed and/or infected. These findings provide data regarding monitoring VL in endemic or silent regions for the disease, can become an additional element in epidemiological and environmental surveillance systems. The high prevalence of anti-*Leishmania* antibodies in these animals may indicate the presence of insect vectors, specifically sandflies, and the circulation of protozoa of the genus *Leishmania*.

In a similar approach, Hamer et al. (2012) demonstrate the presence of multiple zoonotic pathogens using migratory birds as sentinels, providing an assessment of potential public health risks to human populations.

Thus, this type of study may result in an additional tool for health surveillance systems with regard to the monitoring of Leishmaniasis in Brazil.

## Conclusion

Given the results presented in this study, it can be concluded that, although birds have physiological mechanisms that possibly make them refractory to the development of diseases caused by *Leishmania* spp., the blood of these animals is a preferred food source for insect vectors of the subfamily Phlebotominae and contact with these protozoa can induce the production of antibodies. This suggests to us that these animals can be used both as bioindicators for to verify the presence of these insects, as sentinels for the circulation of species of the genus *Leishmania*, becoming an additional tool for health surveillance systems.

However, we consider that further studies using other more specific diagnostic tools, such as PCR, are necessary to deepen the understanding of the relationship between poultry and the epidemiology of leishmaniasis in Brazil.
